# Models for resonant acoustic metasurfaces with application to moth wing ultrasound absorption

**DOI:** 10.1098/rsta.2022.0005

**Published:** 2022-11-28

**Authors:** Yao-Ting Wang, Zhiyuan Shen, Thomas R. Neil, Marc W. Holderied, Elizabeth A. Skelton, Richard V. Craster

**Affiliations:** ^1^ Department of Mathematics, Imperial College London, London SW7 2AZ, UK; ^2^ School of Biological Sciences, University of Bristol, Bristol BS8 1TQ, UK; ^3^ Department of Mechanical Engineering, Imperial College London, London SW7 2AZ, UK

**Keywords:** moth wing, metasurface, spring-mass model, sound absorber, acoustics

## Abstract

Taking as bioinspiration the remarkable acoustic absorption properties of moth wings, we develop a simple analytical model that describes the interaction between acoustic pressure fields, and thin elastic plates incorporating resonant sub-structures. The moth wing is an exemplar of a natural acoustic metamaterial; the wings are deeply subwavelength in thickness at the frequencies of interest, the absorption is broadband and the tiny scales resonate on the moth wing acting in concert. The simplified model incorporates only the essential physics and the scales are idealized to flat rigid rectangular plates coupled via a spring to an elastic plate that forms the wing; all the components are deep-subwavelength at desired frequencies. Based on Fourier analysis, complemented by phenomenological modelling, our theory shows excellent agreement with simulation mimicking the moth-wing structure. Moth wings operate as broadband sound absorbers employing a range of scale sizes. We demonstrate that a random distribution of scale sizes generates a broadband absorption spectrum. To further illustrate the potential of the model, we design a deeply sub-wavelength acoustic counterpart of electromagnetically induced reflectance.

This article is part of the theme issue ‘Wave generation and transmission in multi-scale complex media and structured metamaterials (part 2)’.

## 1. Introduction

There is a strong desire for subwavelength, broadband, acoustic metasurface designs for commercial, urban and industrial uses. Here, we draw upon bioinspiration using the remarkable sound absorption of moth wings. Bats have evolved, over millions of years, a highly sophisticated ultrasonic echolocation system and nocturnal hunting strategy that has forced their primary prey, moths, to evolve various defences. A recent study [1] has shown that a moth species, *Bunaea alcinoe*, has wings equipped with ultrasonic absorbers via a microstructure of wing scales that greatly diminish acoustical reflections (20--160 kHz; absorption coefficient up to 0.72; absorber thickness/λ=1/100) to hinder the bat’s echolocation capability. It has been argued [2] that the wave behaviour of the moth wing is equivalent to an acoustic metasurface; each sub-wavelength moth scale has resonances in the operating frequency range and the thin chitin wing membrane provides not only a vibrating reactive substrate but also links scales on both sides of the wing. Furthermore, a random arrangement of scales in this scale-membrane-scale structure leads to broadband absorbing features whose bandwidth covers most of the ultrasonic frequency range generated by bats. An attractive feature of the moth wing is that it is an ultra-thin (two order of magnitude thinner than the operating wavelength), extremely light (a few tens of milligrams) ultrasonic absorber and we seek to understand this further through the development of a simple acoustic model that can ultimately design absorbers on different scales and for frequency ranges in the audible range.

The field of acoustic metasurfaces has progressed dramatically in recent years and conventionally, acoustic metasurfaces fall into two main categories: those based around resonant cavities, realized by particular configurations with slits [3,4], space-coiling structures [5–11], Helmholtz resonators [12–16] and decorated membrane resonators with sections of thinner or mass-loaded membranes [17–30]. These ideas are leading to broadband diffusers and absorbers [31–33] often based around concepts of grading resonators and so-called rainbow trapping. Tunable or re-configurable acoustic metasurfaces [34] are now viable as are extensions to more complex arrangements of resonators coupled to air gaps [35,36]. The underlying physics of these acoustical metasurfaces are all based on the sonic interaction with either oscillating flow of fluid or vibrating embedded structures. For the moth wing, due to its resonant features, we anticipate that a similar physical interpretation will allow us to model the moth wing via metamaterial perspectives; the main difference is that the scales are exterior to the membrane and not embedded within it.

In the modelling, we want to have generality so that, in the future, a wide range of resonant micro-structures can be considered. We will draw upon impedance patch modelling [37] that had a focus on underwater acoustics. In the sub-wavelength limit, the problem is substantially simplified and idealized to a system consisting of multiple springs and mass loads. Building in their collective behaviour, we provide theoretical predictions of reflection, transmission and absorption, which can all be numerically or experimentally verified. The approach that we use to build the model also facilitates the exploration of novel phenomena based on this ultra-thin structure, paving the way for further scientific investigation.

This paper is organized as follows: in §2, we consider a model for an acoustic metasurface decorated with vibrating plates as a model for a moth wing under the long-wavelength condition, which means the wave scattering caused from the distance between scales and membrane is negligible. By assuming the moth wing to be a periodic structure, an approach based on Fourier analysis is employed for calculating the pressure field throughout the entire space. The derivation involves the vibrations from both spring-mass resonators and thin plate and quantitatively analyses their contribution to ultrasonic waves. In §3a, since the real moth-wing structure naturally contains randomly arranged scales, we add randomness back to the system and its reflection, transmission, and absorption spectra are theoretically determined. In §3b, we study an acoustic metasurface giving perfect transmission across a surface with large impedance mismatch. We gather together concluding remarks in §4.

## 2. Modelling

**Figure 1.**
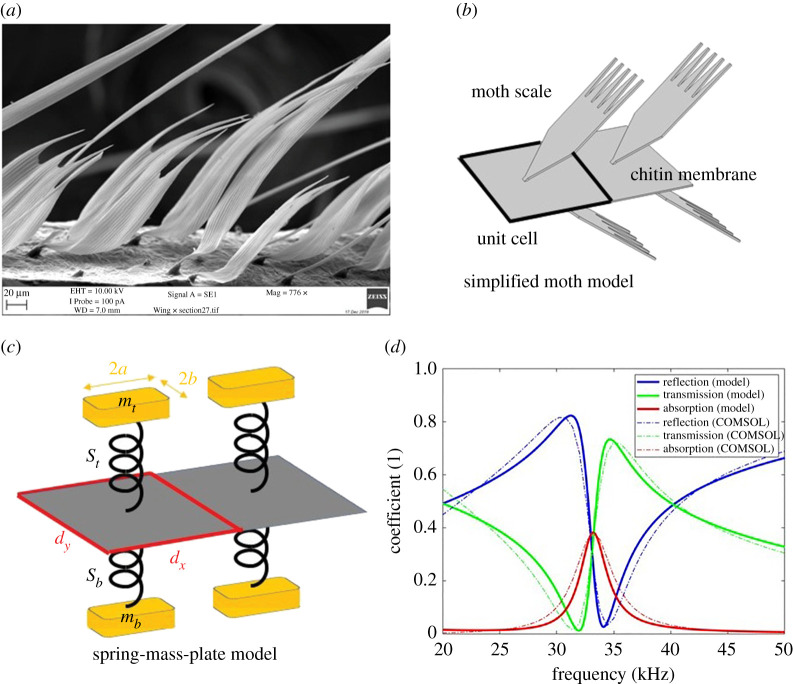
(*a*) A scanning electron microscope (SEM) image for a moth wing showing the scale-membrane structure on the top surface of the wing from an Oak eggar moth (*Lasiocampa quercus*). (*b*) An acoustical metasurface consisting of periodic morphological scales connected on a chitin membrane. (*c*) The idealized spring-mass-plate model based around (*b*). (*d*) The reflection, transmission and absorption spectra calculated from our analytic model (solid lines) and numerical simulations taken from [1] (dashed lines). (Online version in colour.)

Figure 1*a* shows a scanning electron microscopy (SEM) image of a moth wing which consists of a number of chitin scales, placed roughly on a periodic lattice but with some randomness in position and/or scale size [2], with a similar scaling pattern on the other side of the chitin membrane. The scales are non-uniform and contain a variety of shapes that indicate a range of mass loads may be required in the model and motivates the randomness of §3a. Typical scales (shown idealized in figure 1*b*) are roughly 300 μm long from the socket to the tip of the longest apical extension. The blade is roughly triangular with the greatest width at the base of the incisions being 190 μm, and a distance between the tips of the two most distant spines at the top of the scale ranging from 20 to 100 μm; the distance between scales ranges from 150 to 200 μm. [1,2] use experiments and simulation to demonstrate that individual scales vibrate at ultrasonic frequencies having more than one resonance and together they span the entire bat biosonar frequency range. Computations using commercial finite-element software (Comsol) give reflection, transmission and absorption coefficients and these are shown in figure 1*d*.

The moth scales considered in [1] exhibited different modes of resonance. The measured and calculated resonances showed good agreement in mode shape, with the first being a pitch vibration, the second mode a twisting vibration and the third a yaw vibration of the scale [1]. The pitch resonance has an out-of-plane displacement analogous to the idealized scale oscillating up and down and we construct a simplified, and highly idealized model of the system with each scale regarded as a spring-mass resonator as shown in figure 1*c*. Each scale is now a flat rigid rectangle (mass $m_{t}$ or $m_{b}$, with the subscripts t,b corresponding to top or bottom and of sides 2a,2b) parallel to the wing (modelled as an elastic plate). Each rectangle is attached by a spring passing through the plate to an identical rectangle on the other side; in the long wave limit the shape of the ‘scale’ is irrelevant and, if we assume the scale is close to the plate then the gap between them is also irrelevant. The wing is a chitin membrane which is idealized as a thin elastic plate whose interaction with sound is also taken into account as the wing itself reacts to acoustic pressure and vibrates. We model the scale resonator/wing system as this mass-spring-plate idealization and demonstrate that such a model leads to very similar reflection, transmission and absorption coefficients with plausible estimates for the parameters. We begin by assuming that the scales are arranged periodically and later, in §3a, we will incorporate randomness by adding a series of different mass loads generated from a uniform probability density function.

### (a) Non-elastic model

We begin by considering the wing to be a rigid plate that has a periodic array of mass-spring scales on the top and bottom of the plate coupled via a connection of the spring through the plate. The same fluid, air for the moth application, is above and below the plate, and has density ρ and sound speed c. Time-harmonic behaviour at fixed frequency ω is assumed and the wavenumber k=ω/c; exp⁡(−iωt) dependence of the physical fields are henceforth understood and suppressed.

The scales (side lengths 2a,2b) are arranged on a periodic rectangular array and the scales above, and below, the plate have identical areas, $A^{\text{scale}}$, we centre our coordinate system on one of the scales and the elementary cell that contains it; the area of the cell is $A^{\text{cell}}$ (side lengths $d_{x},d_{y}$) and the relative area of scale coverage, A, is $A=A^{\text{scale}}/A^{\text{cell}}$.

We consider an incident plane wave with angles of incidence $\left(\theta_{i},\phi_{i}\right)$ in spherical polar coordinates. The plate lies in the *x*-*y* plane and in z>0 the total pressure field in the upper half-space, $p_{t}$, is separated into an incident and reflected field (as if the plate were rigid and with no scales) and a scattered component $p_{t,b}^{\text{scale}}$ from the scales

2.1 $$p_{t}(x,y,z)=2\exp[-i(\alpha_{i}x+\beta_{i}y)]\cos(\gamma_{ti}z)+p_{t}^{\text{scale}}(x,y,z)$$

and the transmitted field in z<0 (formally z<−h but we assume h≪1)

2.2 $$p_{b}(x,y,z)=p_{b}^{\text{scale}}(x,y,z).$$

Without loss of generality, we take the incoming plane wave to have unit amplitude and the wavevector components are $\alpha_{i}=k\sin\theta_{i}\cos\phi_{i}$, $\beta_{i}=k\sin\theta_{i}\sin\phi_{i}$ and $\gamma_{i}=k\cos\theta_{i}$.

Using Fourier analysis [37], the scattered pressures, in the long-wave limit, are (see appendix A for the detailed derivation)

2.3 $$p_{q}^{\text{scale}}(x,y,z)=\pm \frac{\rho \omega^{2}W_{q}^{\text{scale}}}{A^{\text{cell}}}\sum_{m\in Z}\sum_{n\in Z}\frac{G(\alpha_{m},\beta_{n})}{i\gamma_{mn}}\exp[i(\alpha_{m}x+\beta_{n}y\pm \gamma_{mn}z)]$$

for q=t,b and ± for t,b, respectively. Here, $\alpha_{m}=-(\alpha_{i}+2\pi m/d_{x})$, $\beta_{n}=-(\beta_{i}+2\pi n/d_{y})$, $\gamma_{mn}=\gamma (\alpha_{m},\beta_{n})=\sqrt{k^{2}-\alpha_{m}^{2}-\beta_{n}^{2}}$, $G(\alpha_{m},\beta_{n})=4\sin(\alpha_{m}a)\sin(\beta_{n}b)/\alpha_{m}\beta_{n}$. In the limit of $kd_{x},kd_{y}\to 0,$ we have $G(0,0)∼A^{\text{scale}}$ and $\gamma_{00}=k\cos\theta_{i}$; in the far-field, only the specular reflected wave term propagates and

2.4 $$p_{q}^{\text{scale}}(x,y,z)∼\pm \frac{\rho \omega^{2}AW_{q}^{\text{scale}}}{i\gamma_{i}}\exp[-i(\alpha_{i}x+\beta_{i}y)\pm i\gamma_{i}z]$$

for q=t,b and ± for t,b, and the reflection coefficient, R, follows as:

2.5 $$R∼\left(1+\frac{\rho \omega^{2}AW_{t}^{\text{scale}}}{i\gamma_{i}}\right).$$

Thus we need to identify the displacement of the scale on the upper surface and we do so by balancing forces at the top and bottom scale, $\left(F_{t},F_{b}\right)$, using the forces imposed on the fluid by the scale and those imposed on the scale by the fluid. Doing so leads to a matrix equation for the scale displacements in terms of specific impedances Z, (p=−iωZW), that capture the physics

2.6 $$-i\omega \left[\begin{array}{cc} Z_{11}+A(Z_{a}+Z_{c}) & -Z_{12}\\ -Z_{12} & Z_{22}+A(Z_{a}+Z_{c}) \end{array}\right]\left[\begin{array}{c} W_{t}^{\text{scale}}\\ W_{b}^{\text{scale}} \end{array}\right]=\left[\begin{array}{c} -2\\ 0 \end{array}\right].$$

Here, $Z_{a}$ and $Z_{b}$ are given explicitly in equation (2.9). This equation is obtained since the averaged incident and reflected pressure components, plus that from the displacements of isolated scales and their interactions impose a force on the scale

2.7 $$-\frac{F_{t}}{A^{\text{scale}}}=2-i\omega A(Z_{a}+Z_{c})W_{t}^{\text{scale}}\;\mathrm{and}\;-\frac{F_{b}}{A^{\text{scale}}}=-i\omega A(Z_{a}+Z_{c})W_{b}^{\text{scale}}.$$

This then interacts through a resonant local system that connects forces on the top scale to the lower one and vice-versa

2.8 $$-i\omega \left[\begin{array}{cc} Z_{11} & -Z_{12}\\ -Z_{12} & Z_{22} \end{array}\right]\left[\begin{array}{c} W_{t}^{\text{scale}}\\ W_{b}^{\text{scale}} \end{array}\right]=\frac{1}{A^{\text{scale}}}\left[\begin{array}{c} F_{t}\\ F_{b} \end{array}\right].$$

The specific impedances incorporate the physics, where

2.9 $$-i\omega Z_{a}=\frac{\rho \omega^{2}}{i\gamma_{i}}\;\mathrm{and}\;-i\omega Z_{c}=\rho \omega^{2}\sum_{m\in Z\setminus \{0\}}\sum_{n\in Z\setminus \{0\}}\frac{G^{2}(\alpha_{m},\beta_{n})}{i\gamma_{mn}{\left(A^{\text{scale}}\right)}^{2}}$$

are the specific acoustic impedance for the oscillating scale in the cell ignoring any influence from other scales and the specific impedance correction for the pressure created by the surrounding oscillating scales, respectively. The notation m,n∈Z∖{0} means the summation over all the nonzero integers. The dynamical locally resonant system comprises two masses $m_{t}$ and $m_{b}$ attached individually on a elastic plate by two springs with stiffness $S_{t}$ and $S_{b}$; these are obtained by matching the resonance of the idealized system with experimental and simulation data. Since the scales in a real moth wing are connected with the chitin membrane at a small point, only the force and displacement are considered and the idealized connection of the scales across the membrane pins the connection point to the membrane. According to Newton’s second law of motion and Hooke’s Law, the equations of motion for this spring-mass system are

2.10a $$\begin{array}{rl} & \;-\omega^{2}m_{t}W_{t}^{\text{scale}}+S_{t}(W_{t}^{\text{scale}}-W_{e})=F_{t}, \end{array}$$

2.10b $$\begin{array}{rl} & \;-\omega^{2}m_{b}W_{b}^{\text{scale}}+S_{b}(W_{e}-W_{b}^{\text{scale}})=F_{b} \end{array}$$

2.10c $$\begin{array}{rl} \mathrm{and}\; & \;(S_{t}+S_{b})W_{e}=S_{t}W_{t}^{\text{scale}}+S_{b}W_{b}^{\text{scale}}, \end{array}$$

where $W_{e}$ is the displacement of the connection point. As the desired wavelength range is much larger than the thickness of the plate, the specific impedance $Z_{ij}$ is thus given by eliminating $W_{e}$ as $-i\omega Z_{11,22}=(S_{h}-\omega^{2}m_{t,b})/A^{\text{scale}}$, $-i\omega Z_{12}=S_{h}/A^{\text{scale}}$ and $S_{h}=S_{t}S_{b}/(S_{t}+S_{b})$.

### (b) Elastic plate

Incorporating the elastic plate is a relatively straightforward extension and we modify the pressure fields in (2.1) and (2.2) to have an additional scattered component from the elastic plate, $p_{t}^{\text{elastic}}(x,y,z)$ so in z>0

2.11 $$p_{t}(x,y,z)=2\exp[-i(\alpha_{i}x+\beta_{i}y)]\cos(\gamma_{ti}z)+p_{t}^{\text{scale}}(x,y,z)+p_{t}^{\text{elastic}}(x,y,z)$$

and the transmitted field in z<0 (formally z<−h but we assume h≪1)

2.12 $$p_{b}(x,y,z)=p_{b}^{\text{scale}}(x,y,z)+p_{b}^{\text{elastic}}(x,y,z).$$

The extension relies upon modifying the specific impedances, $Z_{c},Z_{a}$, insofar as they impact upon the displacements of the scales and requiring coupling of the fluid pressure to an elastic plate equation, here an isotropic Kirchhoff–Love plate equation is appropriate due to the long wavelength, for the elastic plate displacement $W_{e}(x,y)$ to the applied pressure on the plate for which

2.13 $$\left[D\left(\frac{\partial^{4}}{\partial x^{4}}+\frac{\partial^{4}}{\partial y^{4}}+2\frac{\partial^{4}}{\partial x^{2}\partial y^{2}}\right)-\omega^{2}\rho_{p}h\right]W_{e}(x,y)=p_{b}(x,y,-h∼0)-p_{t}(x,y,0),$$

where $\rho_{p}$ is the plate density and the $D=Eh^{3}/12(1-\nu^{2})$ is the bending stiffness, E is Young’s modulus and ν the Poisson ratio. Since we are using the notation of specific impedance we introduce the specific plate impedance, $Z_{p}$, in terms of the flexural plate wavenumber, $k_{f}$, as

2.14 $$-i\omega Z_{p}=-\omega^{2}\rho_{p}h\left[1-\frac{{\left(\alpha_{i}^{2}+\beta_{i}^{2}\right)}^{2}}{k_{f}^{4}}\right]=S(\alpha_{i},\beta_{i}),\;\mathrm{where}\;k_{f}^{4}=\frac{\rho_{p}h\omega^{2}}{D},$$

$S(\alpha_{i},\beta_{i})$ is the plate stiffness and the dynamic stiffness incorporating the plate fluid loading is $-i\omega (Z_{p}+2Z_{a})=$ $D(\alpha_{i},\beta_{i})=[D{\left(\alpha_{i}^{2}+\beta_{i}^{2}\right)}^{2}-\omega^{2}\rho_{p}h]-2i\omega^{2}\rho /\gamma (\alpha_{i},\beta_{i})$. The additional elastic plate displacement, $W_{e}$, enters a force balance for which we obtain, from (2.13), that

2.15 $$-i\omega ((Z_{p}+2Z_{a})W_{e}+AZ_{a}[W_{t}^{\text{scale}}+W_{b}^{\text{scale}}])=-2$$

and further using $p_{t}^{\text{elastic}}=-i\omega Z_{a}W_{e}$ and this gives us the extra elastic pressure as (see appendix B for the detailed derivation)

2.16 $$p_{t}^{\text{elastic}}(x,y,z)=\frac{\rho \omega^{2}}{D(\alpha_{i},\beta_{i})}\left[\frac{\rho \omega^{2}v}{\gamma_{i}^{2}}(W_{t}^{\text{scale}}+W_{b}^{\text{scale}})-\frac{2}{i\gamma_{i}}\right]\exp[-i(\alpha_{i}x+\beta_{i}y)+i\gamma_{i}z)].$$

Thence, from (2.11), the reflection coefficient is

2.17 $$R∼\left[1-\frac{2\rho \omega^{2}}{i\gamma_{i}D(\alpha_{i},\beta_{i})}+\rho \omega^{2}A\left(\frac{W_{t}^{\text{scale}}}{i\gamma_{i}}+\frac{\rho \omega^{2}(W_{t}^{\text{scale}}+W_{b}^{\text{scale}})}{D(\alpha_{i},\beta_{i})\gamma_{i}^{2}}\right)\right]$$

and again is determined once the scale displacements are known.

The form of $p_{t}^{\text{scale}}$ is unchanged from (2.4), however the force balances in (2.7) now pick up corrections from the elastic plate and read as

2.18 $$\begin{array}{rl} -\frac{F_{t}}{A^{\text{scale}}} & \;=2\left[1-\frac{\rho \omega^{2}}{i\gamma_{i}D(\alpha_{i},\beta_{i})}\right]-i\omega A(Z_{a}+Z_{c})W_{t}^{\text{scale}}\\ & \;\;-i\omega A(Z_{a}^{\text{elastic}}+Z_{c}^{\text{elastic}})(W_{t}^{\text{scale}}+W_{b}^{\text{scale}}) \end{array}$$

and

2.19 $$\begin{array}{rl} -\frac{F_{b}}{A^{\text{scale}}} & \;=\frac{-2\rho \omega^{2}}{i\gamma_{i}D(\alpha_{i},\beta_{i})}-i\omega A(Z_{a}+Z_{c})W_{b}^{\text{scale}}\\ & \;\;-i\omega A(Z_{a}^{\text{elastic}}+Z_{c}^{\text{elastic}})(W_{t}^{\text{scale}}+W_{b}^{\text{scale}}), \end{array}$$

where the specific impedances [37] are

2.20 $$-i\omega Z_{a}^{\text{total}}=-i\omega (Z_{a}+Z_{a}^{\text{elastic}})=\rho \omega^{2}\left[\frac{1}{i\gamma_{i}}+\frac{\rho \omega^{2}}{\gamma_{i}^{2}D(\alpha_{i},\beta_{i})}\right]$$

and

2.21 $$\begin{array}{rl} -i\omega Z_{c}^{\text{total}} & \;=-i\omega (Z_{c}+Z_{c}^{\text{elastic}})\\ & \;=\rho \omega^{2}\sum_{m\in Z\setminus \{0\}}\sum_{n\in Z\setminus \{0\}}\frac{G^{2}(\alpha_{m},\beta_{n})}{{\left(A^{\text{scale}}\right)}^{2}}\left[\frac{1}{i\gamma (\alpha_{m},\beta_{n})}+\frac{\rho \omega^{2}}{\gamma^{2}(\alpha_{m},\beta_{n})D(\alpha_{m},\beta_{n})}\right]. \end{array}$$

Combining the force balances in (2.18) and (2.19) with the forces from the local resonant system (2.8) gives

2.22 $$\begin{array}{rl} & \;-i\omega \left[\begin{array}{cc} Z_{11}+A(Z_{a}^{\text{total}}+Z_{c}^{\text{total}}) & A(Z_{a}^{\text{elastic}}+Z_{c}^{\text{elastic}})-Z_{12}\\ A(Z_{a}^{\text{elastic}}+Z_{c}^{\text{elastic}})-Z_{12} & Z_{22}+A(Z_{a}^{\text{total}}+Z_{c}^{\text{total}}) \end{array}\right]\left[\begin{array}{c} W_{t}^{\text{scale}}\\ W_{b}^{\text{scale}} \end{array}\right]\\ & \;\;=\left[\begin{array}{c} -2\\ 0 \end{array}\right]+\frac{2\rho \omega^{2}}{i\gamma_{i}D(\alpha_{i},\beta_{i})}\left[\begin{array}{c} 1\\ 1 \end{array}\right] \end{array}$$

which determines the displacements required for the reflection coefficient in (2.17).

## 3. Results

To validate the proposed theory, we use material and geometrical parameters from moth wings using values from [1] where possible. An isotropic thin chitin elastic plate ($\rho_{p}=1300\;\mathrm{kg}\;m^{-3}$, $E=65\times 10^{9}\;\mathrm{Pa}$, ν=0.35 and h=3 μm) between two air spaces ($\rho =1.21\;\mathrm{kg}\;m^{-3},c=343\;m\;s^{-1}$). The plate vibration is excited by a plane wave from the top with an incident angle ($\theta_{i},\phi_{i}$). The mass loads, $m_{t}=m_{b}=2.56\times 10^{-11}\;\mathrm{kg}$, are connected by springs of stiffness $S_{t}=S_{b}=1.2\;N\;m^{-1}$. The mass value is determined by $\rho_{\text{scale}}\times V_{\text{scale}}$ (using values for chitin and dimensions of moth scales) and loss is introduced by adding dynamical loss corrections into the impedance $Z_{11,22}$ in equation (2.10*a*)–(2.10*c*), i.e. $-i\omega Z_{11,22}=S_{t,b}-i\omega \eta_{s}-\omega^{2}m_{t,b}$ with $\eta_{s}=1.2\times 10^{-7}\;\mathrm{kg}\;s^{-1}$. The estimated size of scales is a=b=44 μm, with lattice constant $d_{x}=d_{y}=185\;\mu m$.

Figure 1*d* shows a reflection-transmission-absorption (RTA) spectrum ranging from 20 to 50 kHz. The dash lines present the RTA spectrum from the numerical simulation based one the finite-element method (COMSOL multiphysics 5.6), which serves as a benchmark for the performance of our model. The detailed setting parameters pertaining to this numerical model can be found in [1] except that the scale width is changed to 50 μm. The results from our model and numerical simulation fairly match each other. When the frequency is around 33 kHz, the spectra demonstrate a clear resonant feature and absorption peak for normally incident plane wave; from the symmetry of the resonating system the maximal absorption for this configuration is limited to 0.5.

### (a) Broadband behaviour via randomness

**Figure 2.**
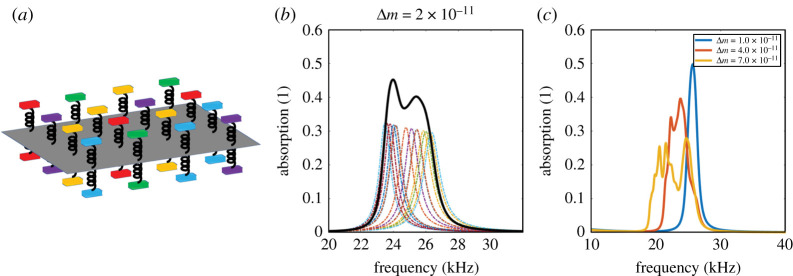
(*a*) A mixed spring-mass-plate model consisting of 4×4 units. Each sub-unit is resonant at different frequency and together they generate the desired broadband absorption feature. (*b*) Absorption peaks from 16 independent randomly distributed mass terms and their overall absorption spectrum after combination. (*c*) Absorption spectra with different deviation strength Δm. The bandwidth expands despite the fact that the absorbing response is lower. (Online version in colour.)

Empirical measurements on real intact moth wings found no regular local randomness patterns in the measured resonance frequencies, but confirm that collective resonances of moth scales are distributed approximately evenly across the biologically relevant frequency range in which acoustic absorption is observed. Moth wings are therefore made up of an array of sub-wavelength resonant elements that span the frequency range required for absorption. The semi-empirical model used in [1] was adapted in [2] to demonstrate that differently tuned scales arranged in a pandiagonal magic square could work together to create broadband deep sub-wavelength sound absorption, confirming moth wings as a naturally occurring acoustic metamaterial. The absorption spectra for moth wings cover a wide range of frequencies, and we now consider how we can achieve broadband behaviour within the model; the SEM images of the scales figure 1*a* show randomness in the size and position of the scales. To replicate the biological randomness, we now introduce randomness into the spring-mass model noting that the pressure in the entire space is obtained when the displacements $W_{t}^{\text{scale}}$ and $W_{b}^{\text{scale}}$ are both known. In figure 2*a*, we consider a multi-component composite made of N spring-mass resonators that are then periodically extended to cover the wing. To introduce randomness, the magnitude of each mass load is assumed to be a perturbation around a fixed mass $m_{t(b)}^{\left(0\right)}$

3.1 $$m_{t(b)}^{\left(i\right)}=m_{t(b)}^{\left(0\right)}+\Delta m_{t(b)}R(i),$$

where R(i) (i=1,2,…,N) are random numbers generated from uniform probability density function distributed on [0,1]; each mass load gives a specific displacement $W_{t(b)}^{\text{scale}(i)}$. We then take the displacement for the entire unit cell (the 4×4 grouping in the example shown in figure 2*a*, i.e. N=16) is replaced by their average, $\left\langle W_{t(b)}^{\text{scale}}\right\rangle$, as

3.2 $$\langle W_{t(b)}^{\text{scale}}\rangle =\frac{\left(W_{t(b)}^{\text{scale}(1)}+W_{t(b)}^{\text{scale}(2)}+\cdots +W_{t(b)}^{\text{scale}(N)}\right)}{N}$$

assuming the long-wavelength condition holds for this super-cell structure. After substituting $\left\langle W_{t(b)}^{\text{scale}}\right\rangle$ for $W_{t(b)}^{\text{scale}}$ in §3 the pressure fields in the entire space are determined as are the reflection coefficients. We take both mass terms to range between $7\times 10^{-11}$ and $9\times 10^{-11}\;\mathrm{kg}$ with the fixed mass terms $m_{t,b}^{0}$ both set as $7\times 10^{-11}$ and the deviation strengths $\Delta m_{t,b}$ are $2\times 10^{-11}$. To more clearly demonstrate the effect resulting from deviation strengths, the unit cell parameters a and b are both changed to be 50 μm, and the material parameter $S_{t,b}$ and $\eta_{s}$ are changed to be $1.02\;N\;m^{-1}$ and $1\times 10^{-7}\;\mathrm{kg}\;s^{-1}$, respectively. Figure 2*b* demonstrates a broadband absorption spectrum in a 4×4 resonator array. The bandwidth is significantly wider compared with the one from a single unit cell. We investigate the effect of $\Delta m_{t,b}$ by additionally using the values $1\times 10^{-11}$, $4\times 10^{-11}$ and $7\times 10^{-11}\;\mathrm{kg}$ to show that the absorption bandwidth can be increased when $\Delta m_{t,b}$ is increased; figure 2*c* demonstrates the broadening of the absorption bandwidth, although notably the absorption peak is flattened. A moth wing contains thousands of different scales, and this variation assists in making it an efficient ultrasound absorber whose absorbing bandwidth spans the ultrasonic frequency range generated by bats [2].

### (b) Air-to-water perfect transmission

We have shown that the idealized analytical model enables us to consider broadband absorbers. We now move to a device outside the biological motivation and investigate perfect transmission across an air/water interface at audible frequencies. Due to the large impedance mismatch between air and water ($\rho_{\text{water}}=1000\;\mathrm{kg}\;m^{-3}$, $c_{\text{water}}=1490\;m\;s$, $Z_{\text{water}}/Z_{\text{air}}\approx 3600$), most sound energy will naturally be reflected. Yet, a recent study has shown that air-to-water perfect transmission occurs when there is a sonic metasurface made of a set of membrane resonators and an air cavity [30]. For such structure, the air-to-water impedance ratio is unity but the corresponding imaginary part approaches zero at a certain frequency. Despite the great improvement of sound transmission, their design requires a fine control on the membrane’s initial stresses, which is sensitive to the resonant frequency. In the following, we discuss the air-to-water transmission based on our bioinspired structure. This style of design has advantages because it does not consist of any pre-tensioned membranes and cavities, thereby making fabrication easier to implement owing to the development of three-dimensional printing technology.

**Figure 3.**
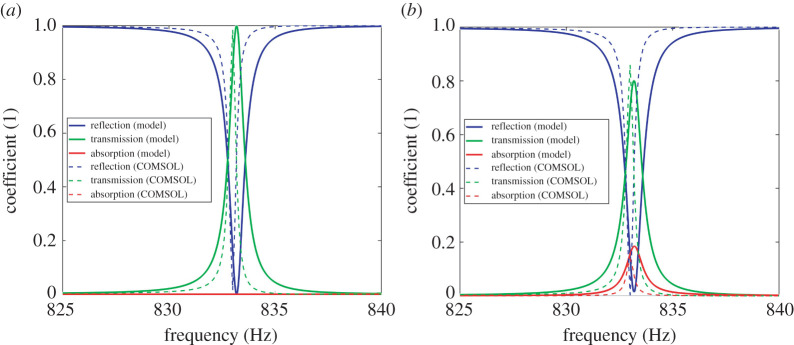
(*a*) A perfect-transmission effect in an acoustic metasurface designed by the idealized theory (solid line) and verified by COMSOL simulation (dash line). When the frequency is around 833 Hz, the transmission reaches 100%, leading to a nearly perfect sonic transmission. (*b*) The RTA spectra of the perfection-transmission effect with viscoelasticity factor $\eta_{s}=10\;\mathrm{kg}\;s^{-1}$. It shows an 80% transmission peak for the proposed model and numerical simulation. (Online version in colour.)

We consider a new lossless model with material and geometrical parameters chosen as follows: a=b=120 mm, $d_{x}=d_{y}=180\;\mathrm{mm}$, h=4 mm, $m_{t}=m_{b}=0.26\;\mathrm{kg}$, $S_{t}=S_{b}=1\;N\;m^{-1}$. Also, the material of the thin elastic plate is epoxy ($\rho_{p}=1200\;\mathrm{kg}\;m^{-3}$, $E=3.5\times 10^{9}\;\mathrm{Pa}$, ν=0.33). Regarding the COMSOL simulation, the material and geometrical parameters of thin plate are the same as the analytical model. For the simplified scale, the width and length of the shaft are 11 mm and 61 mm, and the corresponding geometry parameters for the blade are 120 mm and 160 mm. With these parameters, in figure 3*a*, we illustrate two RTA spectra from theory and COMSOL clearly showing a perfectly transmitted characteristic around 833 Hz. Both transmission spectra rapidly rise up to nearly one, whereas the reflection spectrum exhibits a sharp dip. Moreover, figure 3*b* demonstrates RTA spectra with realistic loss factors $\eta_{s}=10\;\mathrm{kg}\;s^{-1}$ for the analytical model, Kelvin–Voigt viscoelastic parameter $\eta_{\text{KG}}=10\;\mathrm{kg}\;s^{-1}$ for COMSOL simulation. Although the absorption peak rises, a strong transmission peak remains, thereby suggesting air-to-water perfect transmission is practical.

## 4. Conclusion

In conclusion, an analytic model that describes the vibration of moth wings has been proposed which creates a model metasurface decorated with mass-spring oscillators; in this article, we concentrate upon a simple resonant oscillator system whereas the real moth wing consists of multiple scales layers of different sizes, shapes and degrees of overlap. A scale’s morphology determines its resonances, and the diversity of scale morphologies is reflected in the scale resonance distribution [2]. Furthermore, moth scales show multiple mode shapes which all contribute to the broadband absorptive performance [1]. The idealized model presented here is limited to the out-of-plane displacements, but is quite general in terms of the resonant objects that can be modelled and the local resonant system represented through the forces and displacements in (2.8) can be easily generalized to more complex systems. Studying the RTA spectra, and the agreement between the results given by analytical and numerical calculations, gives validation of the proposed model. The advantage of an analytic model is that additional physics, such as randomness, can be incorporated and the bandwidth absorption spectrum expands as the deviation strength increases, which helps explain the broadband absorption that features heavily in experiments on moth wings [1]. Lastly, we look outside the moth motivation and explore the potential of acoustic metasurfaces based around oscillators attached to the surface by investigating an EIR-like effect; the sharp reflection peak, robust against randomness, can be employed to develop acoustical filtering or sensing devices.

## Data Availability

The data are provided in the electronic supplementary material [38].

## Appendix A. Derivation of scale pressure

To derive equation (2.3), the following identities hold when F(x,y) is a periodic function:

A 1 $$\begin{array}{rl} & F(x+md_{x},y+md_{y})=F(x,y)\exp[-i(m\alpha_{i}d_{x}+n\beta_{i}d_{y})]\\ & \;\sum_{m,n\in Z}F(md_{x},md_{y})\exp[-i(m\alpha d_{x}+n\beta d_{y})]\\ & \;\;=\frac{1}{d_{x}d_{y}}\sum_{m,n\in Z}\bar{F}\left(\alpha +\frac{2m\pi }{d_{x}},\beta +\frac{2n\pi }{d_{y}}\right)\\ & \;\sum_{m,n\in Z}\exp(-i[md_{x}(\alpha +\alpha_{i})+nd_{y}(\beta +\beta_{i})])\\ & \;\;=\frac{4\pi^{2}}{d_{x}d_{y}}\sum_{m,n\in Z}\delta \left(\alpha +\alpha_{i}+\frac{2m\pi }{d_{x}}\right)\delta \left(\beta +\beta_{i}+\frac{2n\pi }{d_{y}}\right) \end{array}$$

A 4 $$\begin{array}{rl} & \bar{F}(\alpha ,\beta )=\iint_{-\infty }^{+\infty }F(x,y)\exp[-i(\alpha x+\beta y)]\;dx\;dy:\text{(Fourier transform) } \end{array}$$

A 5 $$\begin{array}{rl} & F(x,y)=\frac{1}{4\pi^{2}}\iint_{-\infty }^{+\infty }\bar{F}(\alpha ,\beta )\exp[i(\alpha x+\beta y)]\;d\alpha \;d\beta :\text{(Inverse Fourier transform) } \end{array}$$

Formulate that relate spectral pressure to top and bottom scale spectral displacements are

A 6 $${\bar{p}}_{t}^{\text{scale}}(\alpha ,\beta ,z)=\rho \omega^{2}\left[\frac{{\bar{W}}_{t}^{\text{scale}}(\alpha ,\beta )}{i\gamma }\right]\exp(i\gamma z)$$

and

A 7 $${\bar{p}}_{b}^{\text{scale}}(\alpha ,\beta ,z)=-\rho \omega^{2}\left[\frac{{\bar{W}}_{b}^{\text{scale}}(\alpha ,\beta )}{i\gamma }\right]\exp[i\gamma (z+h)].$$

As $W_{t}^{\text{scale}}(x,y)$ is periodic and occupies a certain area 2a×2b, based on Bloch’s theorem

A 8 $$\begin{array}{rl} {\bar{W}}_{t}^{\text{scale}}(\alpha ,\beta ) & \;=\sum_{m,n\in Z}\exp[-i(md_{x}\alpha +nd_{y}\beta )]\int_{-a}^{+a}\int_{-b}^{+b}W_{t}^{\text{scale}}(x+md_{x},y+nd_{y})\\ & \;\;\times \exp[-i(\alpha x+\beta y)]\;dx\;dy. \end{array}$$

On using the relations in equation (A 1),

A 9 $$\begin{array}{rl} {\bar{W}}_{t}^{\text{scale}}(\alpha ,\beta ) & \;=\sum_{m,n\in Z}\exp[-i(md_{x}(\alpha +\alpha_{i})+nd_{y}(\beta +\beta_{i}))]\int_{-a}^{+a}\int_{-b}^{+b}W_{t}^{\text{scale}}(x,y)\\ & \;\;\times \exp[-i(\alpha x+\beta y)]\;dx\;dy. \end{array}$$

The displacement of the upper surface is homogenized as incident wavelength is much longer than the periodicity, i.e. $W_{t}^{\text{scale}}(x,y)\approx W_{t}^{\text{scale}}$, so that the spectral displacement ${\bar{W}}_{t}^{\text{scale}}(\alpha ,\beta )$ reads

A 10 $${\bar{W}}_{t}^{\text{scale}}(\alpha ,\beta )=W_{t}^{\text{scale}}G(\alpha ,\beta )\sum_{m,n\in Z}\exp[-i(md_{x}(\alpha +\alpha_{i})+nd_{y}(\beta +\beta_{i}))],$$

where G(α,β)=4sin⁡(αa)sin⁡(βb)/αβ. The identity in equation (A 2) allows the sum of exponentials to be replaced by a sum of delta functions, which facilitates the calculation of the pressure $p_{t}^{\text{scale}}$ as

A 11 $$\begin{array}{rl} {\bar{W}}_{t}^{\text{scale}}(\alpha ,\beta ) & \;=W_{t}^{\text{scale}}G(\alpha ,\beta )\sum_{m,n\in Z}\exp[-i(md_{x}(\alpha +\alpha_{i})+nd_{y}(\beta +\beta_{i}))]\\ & \;=\left(\frac{4\pi^{2}W_{t}^{\text{scale}}}{d_{x}d_{y}}\right)G(\alpha ,\beta )\sum_{m,n\in Z}\delta \left(\alpha +\alpha_{i}+\frac{2\pi m}{d_{x}}\right)\delta \left(\beta +\beta_{i}+\frac{2\pi n}{d_{y}}\right) \end{array}$$

Substituting equation (A 11) into equation (A 6), one obtains

A 12 $$\begin{array}{rl} p_{t}^{\text{scale}}(x,y,z) & \;=\left(\frac{\rho \omega^{2}}{4\pi^{2}}\right)\iint_{-\infty }^{+\infty }\left[\frac{{\bar{W}}_{t}^{\text{scale}}(\alpha ,\beta )}{i\gamma }\right]\exp[i(\alpha x+\beta y+\gamma z)]\;d\alpha \;d\beta \\ & \;=\frac{\rho \omega^{2}W_{t}^{\text{scale}}}{d_{x}d_{y}}\sum_{m,n\in Z}\iint_{-\infty }^{+\infty }\frac{G(\alpha ,\beta )}{i\gamma }\delta \left(\alpha +\alpha_{i}+\frac{2\pi m}{d_{x}}\right)\delta \left(\beta +\beta_{i}+\frac{2\pi n}{d_{y}}\right)\\ & \;\;\times \exp[i(\alpha x+\beta y+\gamma z)]\;d\alpha \;d\beta \\ & \;=\frac{\rho \omega^{2}W_{t}^{\text{scale}}}{d_{x}d_{y}}\sum_{m,n\in Z}\frac{G(\alpha_{m},\beta_{n})}{i\gamma_{mn}}\exp[i(\alpha_{m}x+\beta_{n}y+\gamma_{mn}z)]. \end{array}$$

Equation (A 12) is the final result required for equation (2.3); in a similar manner, $p_{b}^{\text{scale}}$ can be derived.

## Appendix B. Derivation of elastic pressure

To derive equation (2.16), we start with

B 1 $$\left[D\left(\frac{\partial^{4}}{\partial x^{4}}+\frac{\partial^{4}}{\partial y^{4}}+2\frac{\partial^{4}}{\partial x^{2}\partial y^{2}}\right)-\omega^{2}\rho_{p}h\right]W_{e}(x,y)=p_{b}(x,y,-h)-p_{t}(x,y,0).$$

The spectral equation is obtained by applying Fourier transform equation (A 4) to equation (B 1) to get

B 2 $$\begin{array}{rl} S(\alpha ,\beta ){\bar{W}}_{e}(\alpha ,\beta ) & \;=-2(4\pi^{2})P_{0}\delta (\alpha +\alpha_{i})\delta (\beta +\beta_{i})-[{\bar{p}}_{t}^{\text{scale}}(\alpha ,\beta ,0)-{\bar{p}}_{b}^{\text{scale}}(\alpha ,\beta ,-h)]\\ & \;\;-[{\bar{p}}_{t}^{\text{elastic}}(\alpha ,\beta ,0)-{\bar{p}}_{b}^{\text{elastic}}(\alpha ,\beta ,-h)], \end{array}$$

where $S(\alpha ,\beta )=D{\left(\alpha^{2}+\beta^{2}\right)}^{2}-\omega^{2}\rho_{p}h$ is the isotropic spectral dynamic stiffness of the plate. Since the vibrations of the thin plate on the upper and lower surfaces are both equal to ${\bar{W}}_{e}$, the relations in equations (A 6) and (A 7) are valid for the spectral pressures ${\bar{p}}_{t}^{\text{elastic}}(\alpha ,\beta ,0)$ and ${\bar{p}}_{b}^{\text{elastic}}(\alpha ,\beta ,-h)$ and spectral displacement ${\bar{W}}_{e}$, i.e.

B 3 $${\bar{p}}_{t}^{\text{elastic}}(\alpha ,\beta ,0)=\rho \omega^{2}\left[\frac{{\bar{W}}_{e}(\alpha ,\beta )}{i\gamma }\right]\exp(i\gamma z)$$

and

B 4 $${\bar{p}}_{b}^{\text{elastic}}(\alpha ,\beta ,-h)=-\rho \omega^{2}\left[\frac{{\bar{W}}_{e}(\alpha ,\beta )}{i\gamma }\right]\exp[i\gamma (z+h)].$$

Plugging equations (B 3) and (B 4), (A 11) and the corresponding expression for ${\bar{W}}_{b}^{\text{scale}}$ into equation (B 2), one obtains

B 5 $$\begin{array}{rl} D(\alpha ,\beta ){\bar{W}}_{e}(\alpha ,\beta ) & \;=-2(4\pi^{2})P_{0}\delta (\alpha +\alpha_{i})\delta (\beta +\beta_{i})\\ & \;\;-2\left(\frac{4\pi^{2}G(\alpha ,\beta )}{d_{x}d_{y}}\right)\left[\frac{-i\omega^{2}\rho (W_{t}^{\text{scale}}+W_{b}^{\text{scale}})}{\gamma }\right]\\ & \;\;\times \sum_{mn}\delta \left(\alpha +\alpha_{i}+\frac{2\pi m}{d_{x}}\right)\delta \left(\beta +\beta_{i}+\frac{2\pi n}{d_{y}}\right). \end{array}$$

where $D(\alpha ,\beta )=S(\alpha ,\beta )-2i\omega^{2}\rho /\gamma$. Using equations (B 5) and (A 5) gives the pressure in the upper space from elastic vibrations as

B 6 $$\begin{array}{rl} p_{t}^{\text{elastic}}(x,y,z) & \;=\left(\frac{\rho \omega^{2}}{4\pi^{2}}\right)\iint_{-\infty }^{+\infty }\;d\alpha \;d\beta \left[\frac{{\bar{W}}_{e}(\alpha ,\beta )}{i\gamma }\right]\exp[i(\alpha x+\beta y+\gamma z)]\\ & \;=\left(\frac{\rho \omega^{2}}{4\pi^{2}}\right)\iint_{-\infty }^{+\infty }\;d\alpha \;d\beta \{\exp\left[\frac{i(\alpha x+\beta y+\gamma z)}{i\gamma }\right]-\frac{2(4\pi^{2})P_{0}\delta (\alpha +\alpha_{i})\delta (\beta +\beta_{i})}{D(\alpha ,\beta )}\\ & \;\;+\left[\frac{4\pi^{2}G(\alpha ,\beta )}{d_{x}d_{y}D(\alpha ,\beta )}\right]\left[\frac{-i\omega^{2}\rho (W_{t}^{\text{scale}}+W_{b}^{\text{scale}})}{\gamma }\right]\\ & \;\times \sum_{m,n\in Z}\delta \left(\alpha +\alpha_{i}+\frac{2\pi m}{d_{x}}\right)\delta \left(\beta +\beta_{i}+\frac{2\pi n}{d_{y}}\right)\}\\ & \;=\frac{-2P_{0}\rho \omega^{2}}{i\gamma_{i}D(\alpha_{i},\beta_{i})}\exp[-i(\alpha_{i}x+\beta_{i}y+\gamma_{i}z)]\\ & \;\;+\left(\frac{\rho \omega^{4}}{d_{x}d_{y}}\right)\sum_{m,n\in Z}\frac{\rho (W_{t}^{\text{scale}}+W_{b}^{\text{scale}})}{\gamma_{mn}^{2}}\\ & \;\;\times \frac{G(\alpha_{m},\beta_{n})}{D(\alpha_{m},\beta_{n})}\exp[i(\alpha_{m}x+\beta_{n}y+\gamma_{mn}z)]. \end{array}$$

Equation (B 6) is our final result which is simplified to (2.16) in the long wave limit; in a similar manner, the pressure field $p_{b}^{\text{elastic}}(x,y,z)$ can be found.
